# Fluctuations in airway bacterial communities associated with clinical states and disease stages in cystic fibrosis

**DOI:** 10.1371/journal.pone.0194060

**Published:** 2018-03-09

**Authors:** Lisa A. Carmody, Lindsay J. Caverly, Bridget K. Foster, Mary A. M. Rogers, Linda M. Kalikin, Richard H. Simon, Donald R. VanDevanter, John J. LiPuma

**Affiliations:** 1 Department of Pediatrics and Communicable Disease, University of Michigan Medical School, Ann Arbor, Michigan, United States of America; 2 Department of Internal Medicine, University of Michigan, Ann Arbor, Michigan, United States of America; 3 Department of Pediatrics, Case Western Reserve University School of Medicine, Cleveland, Ohio, United States of America; Lee Kong Chian School of Medicine, SINGAPORE

## Abstract

Bacteria that infect the airways of persons with cystic fibrosis (CF) include a group of well-described opportunistic pathogens as well as numerous, mainly obligate or facultative anaerobic species typically not reported by standard sputum culture. We sequenced the V3-V5 hypervariable region of the bacterial 16S rRNA gene in DNA derived from 631 sputum specimens collected from 111 CF patients over 10 years. We describe fluctuations in the relative abundances of typical CF pathogens, as well as anaerobic species, in relation to changes in patients’ clinical state and lung disease stage. Both bacterial community diversity and the relative abundance of anaerobes increased during exacerbation of symptoms (prior to antibiotic treatment), although this trend was not observed uniformly across disease stages. Community diversity and the relative abundance of anaerobic species decreased during antibiotic treatment. These results support current hypotheses regarding the role of anaerobes in CF pulmonary exacerbations and lung disease progression.

## Introduction

The airways of persons with cystic fibrosis (CF) typically harbor diverse, polymicrobial communities comprised of well-described “typical CF pathogens” (*Pseudomonas*, *Achromobacter*, *Burkholderia*, *Haemophilus*, *Staphylococcus*, and *Stenotrophomonas*), as well as a wide variety of other taxa, including many obligate and facultative anaerobic species [1–4]. We recently described the bacterial communities found in a large set of CF sputum specimens by DNA sequencing, and compared these to the taxa reported in these same samples based on conventional CF sputum culture [5]. Not surprisingly, DNA sequencing analysis revealed diverse segments of these communities that were not reported by routine culture. The unreported fraction varied widely from sample to sample, both in terms of richness (the number of different taxa observed) and the relative abundances of the species present. How these variations in bacterial community structure relate to changes in patient clinical condition was not described in that report.

The proposed climax-attack model of CF lung infection is grounded in ecological principals and describes two functional microbial communities in CF airways [6]. The “climax” community is associated with baseline/stable clinical states, while the “attack” community dominates during exacerbations of symptoms. Although these communities are not defined taxonomically *per se*, network analyses have shown that climax communities are comprised primarily of typical CF pathogens, while attack communities are associated with anaerobic species such as *Prevotella*, *Veillonella*, *Fusobacterium*, and microaerophilic *Streptococcus* spp. [7]. With the framework of the climax-attack model in mind, we investigated our data set with regard to the prevalence and relative abundances of typical CF pathogens and anaerobic genera in relation to patient *clinical state*. We also assessed bacterial community structures relative to patient lung *disease stage* and *disease aggressiveness* phenotype.

## Methods

### Patients, sputum specimens and medical record review

The sputum samples and DNA sequences analyzed in this study were a subset of the 945 samples from 132 CF patients we described in a previous report [5]. From this larger data set, we identified 631 samples from 111 patients for whom associated clinical data were available at the time of sample collection (described below).

All sputum samples had been collected during routine medical care and processed by the Michigan Medicine clinical microbiology laboratory. Samples were held at 4**°**C from within 12 hours of collection until they were stored in 0.5mL aliquots, without additional processing, at -80**°**C. Sputum sample collection and medical record review were approved by the University of Michigan Institutional Review Board.

Medical records were reviewed to identify samples that were obtained when the patient’s *clinical state* at the time of sample collection could be determined. Clinical state was defined as previously described [8,9]: *B*, baseline; *E*, pulmonary exacerbation before antibiotic administration; *T*, antibiotic treatment of pulmonary exacerbation; or *R*, recovering from pulmonary exacerbation, within 21 days after cessation of antibiotics prescribed for exacerbation. These samples were further categorized by *disease stage* [8], based on percent predicted forced expiratory volume in one second (%FEV_1_) values on the day of sample collection: *early* (%FEV_1_ > 70), *intermediate* (%FEV_1_ ≤ 70, ≥ 40), or *advanced* (%FEV_1_ < 40). Clinical state (*B*,*E*,*T*,*R*) and disease stage (%FEV_1_) data were available for 631 samples, collected from 111 patients (range 1–24 samples per patient).

We also assessed the impact of *disease aggressiveness* phenotype on bacterial community structures over time in a subset of 24 patients, each of whom had contributed at least 10 sputum samples over at least five years of observation. Disease aggressiveness phenotype (mild, moderate, or severe) was determined based on rate of change of FEV_1_ relative to age using all available FEV_1_ measurements as previously described [10,11]. When a patient’s trajectory of FEV_1_ measurements crossed between two categories, the category with the majority of the measurements was used to determine aggressiveness phenotype (S1 Fig).

### DNA extraction, sequencing and data analyses

DNA was prepared from frozen sputum and bacterial 16S ribosomal subunit (16S rRNA) genes were sequenced as part of a study we described previously [5,8]. Details are available in [5,8] and in the supporting information section [S1 Methods]. Original sff files with metadata are deposited in NCBI Sequence Read Archive (NCBI BioProject ID PRJNA423040).

For sequence analysis of the 631 samples included in the present study, the mothur (v.1.29) software package was used following the standard operating procedures (https://www.mothur.org/wiki/454_SOP). The total number of reads for each sample was rarefied (an average of 1000 subsampling iterations, rounded to the nearest whole number) to 547, the smallest number of reads obtained in the sample set, to control for differences in sequencing depth before alpha diversity measures were calculated. The dominant genus was defined as the most abundant genus observed in the sample. Prevalence was calculated as the number of samples with nonzero relative abundance divided by the total sample size. Non-parametric Shannon dissimilarity (Shannon diversity) was used to describe community richness and evenness [12].

OTUs representing *Pseudomonas*, *Achromobacter*, *Burkholderia*, *Haemophilus*, *Staphylococcus*, and *Stenotrophomonas* were categorized as “typical CF pathogens.” OTUs representing the obligate anaerobic genera *Actinomyces*, *Fusobacterium*, *Porphyromonas*, *Prevotella*, and *Veillonella*, and the facultative anaerobic genera *Gemella*, *Granulicatella*, *Rothia*, and *Streptococcus* were collectively referred to as “anaerobic genera.” Each of these nine anaerobic genera accounted for at least 1% mean relative abundance when present. Collectively, the six typical CF pathogen genera and nine anaerobic genera represented 94.3% of all sequencing reads (60.7% of reads represented typical CF pathogens; 33.6% represented anaerobic genera); the remaining 5.7% of DNA sequencing reads represented other genera.

To accommodate the longitudinal design of this study (89 subjects contributed more than one sample), generalized estimating equations (GEE) were used, with a gamma probability distribution, log link function, and unstructured working correlation matrix. Alpha was set at 0.05 (2-tailed) and analyses were conducted in SPSS (v24).

## Results

### Patient demographics and taxa prevalence by patient age, clinical state, and disease stage

Patient demographics and characteristics are described in Table 1. Trends in the prevalence of the six typical CF pathogens, as well as the nine anaerobic genera, were calculated by patient age, clinical state, and disease stage (S2 Fig). With the exception of *Pseudomonas*, the prevalence of most genera remained steady or dropped with advancing age. Although consistent trends in species prevalence by clinical state were not found, anaerobic genera prevalence decreased at advanced disease stage. However, an increase in the prevalence of some anaerobic genera (*Veillonella*, *Fusobacterium*, *Porphyromonas*) was observed in the 45+ age group (S2B Fig).

**Table 1:** Subject demographics and sample characteristics.

		Subjects	Samples*
		n = 111 (%)	n = 631 (%)
Sex	Male	62 (55)	
CFTR Genotype	F508del / F508del	49 (44)	
	F508del / other	44 (40)	
	other / other	18 (16)	
Mean age, years (range)			26 (6–53)
Clinical State	**B**aseline		268 (42)
	**E**xacerbation		127 (20)
	**T**reatment		113 (18)
	**R**ecovery		123 (19)
Disease Stage	Early (FEV_1_ > 70)		197 (31)
	Intermediate (40 ≤ FEV_1_ ≤ 70)		266 (42)
	Advanced (FEV_1_ < 40)		168 (27)

Metadata associated with subjects and samples.

*clinical metric at the time of sample collection

### Bacterial community changes associated with clinical states and disease stages

At early and advanced disease stages, *Pseudomonas* was the dominant taxon in more than half of the samples (S3 Fig). With the exception of *Actinomyces*, each of the nine anaerobic genera was the dominant taxon in at least one sample, with *Streptococcus* and *Prevotella* being most commonly seen among this group. The proportion of samples in which an anaerobic species was the dominant taxon decreased from ~30% to ~12% with advancing disease stage.

**Fig 1:**
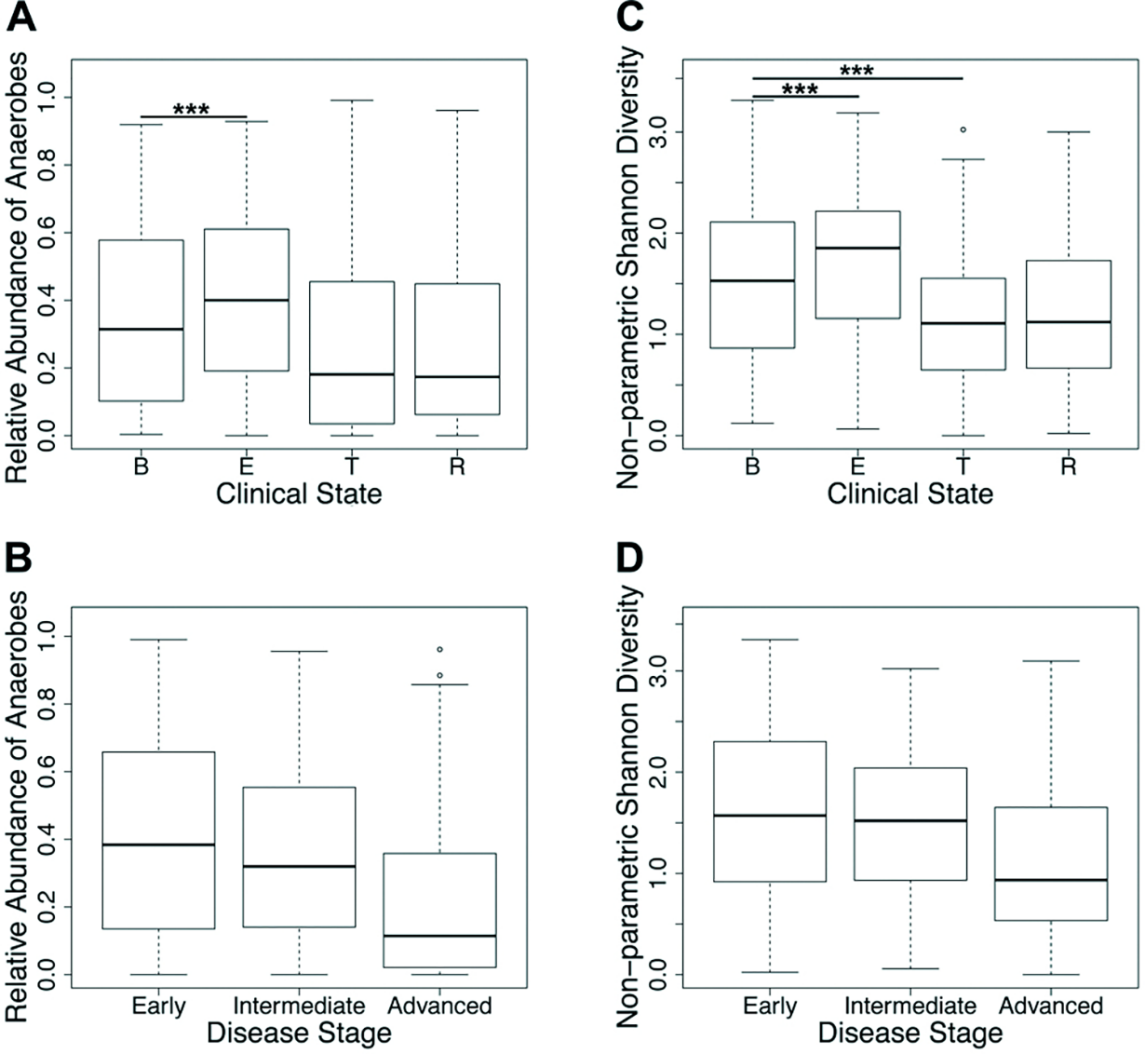
Relative abundance of anaerobic genera and Shannon diversity in bacterial communities based on patient clinical state and disease stage. The cumulative relative abundances of anaerobic genera (*Actinomyces*, *Fusobacterium*, *Gemella*, *Granulicatella*, *Porphyromonas*, *Prevotella*, *Rothia*, *Streptococcus* and *Veillonella* spp) are depicted by (A) clinical state and (B) disease stage. Bacterial community Shannon diversity is depicted by (C) clinical state and (D) disease stage. ***P ≤ 0.001 (GEE).

The cumulative relative abundance of anaerobic genera increased significantly during exacerbation of symptoms prior to episodic antibiotic treatment (*E*) relative to baseline (*B*), and was decreased during treatment (*T*) and recovery (*R*) (Fig 1A). The relative abundance of anaerobic genera declined with advancing disease stage (Fig 1B). Similar trends in Shannon diversity were observed across clinical state and disease stage categories (Fig 1C & 1D).

Generalized estimating equations were used to determine the influence of patient-specific factors on bacterial community structure, while accounting for non-independence of multiple measures collected from the same patient (S1 Table). Both the cumulative relative abundance of anaerobic genera and Shannon diversity were significantly greater in exacerbation samples compared to baseline samples (*P* < 0.001 for both measures). Shannon diversity was also significantly lower for treatment samples compared to baseline samples (*P* < 0.001). Recovery samples were similar to baseline samples in the relative abundance of anaerobic genera and Shannon diversity (*P* > 0.05 for both). Patient lung function was positively associated with cumulative relative abundance of anaerobic genera and with Shannon diversity (*P* < 0.001 for both); greater lung function had significantly higher measures of both metrics. Patient age was not found to have a significant impact on relative abundance of anaerobes (*P* > 0.05), yet was negatively correlated with Shannon diversity (*P* = 0.006).

**Fig 2:**
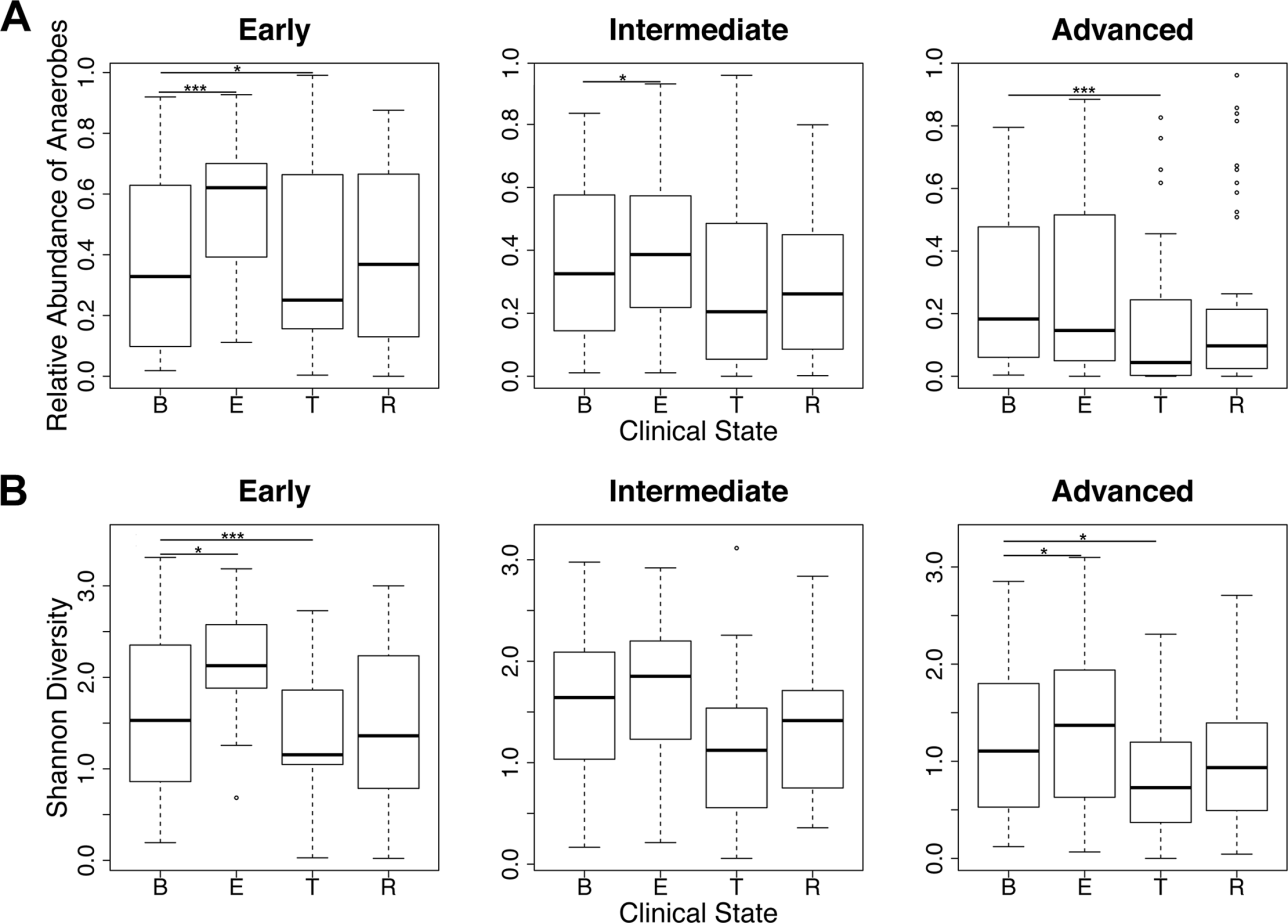
Changes in community structure across clinical states differ based on patient disease stage. (A) Cumulative relative abundance of anaerobic genera (*Actinomyces*, *Fusobacterium*, *Gemella*, *Granulicatella*, *Porphyromonas*, *Prevotella*, *Rothia*, *Streptococcus* and *Veillonella* spp) and (B) Shannon diversity by subject clinical state, at early, intermediate, and advanced lung disease stages.* *P* ≤ 0.05 (GEE), ***P ≤ 0.001 (GEE).

Uniform relationships between patient clinical state and bacterial community structure were not observed across all lung disease stages. The generalized estimating equations described above were repeated for subsets of the data representing each disease stage. The relative abundance of anaerobic genera at exacerbation was significantly higher compared with baseline during early and intermediate disease stages only (Fig 2A, *P* < 0.001 and *P* < 0.017, respectively). At advanced disease stage, the median relative abundance of anaerobic genera was *lower* at exacerbation than at baseline. Shannon diversity was significantly higher at exacerbation during early and advanced disease stages (Fig 2B, *P* < 0.001 for both). Anaerobe relative abundance and Shannon diversity were significantly lower during treatment compared with baseline at early (Fig 2, *P* = 0.017 and *P* <0.001, respectively) and advanced (Fig 2, *P* <0.001 and *P* = 0.003, respectively) disease stages.

### Bacterial community changes associated with disease aggressiveness

Long-term samples sets were available for 24 patients, wherein 10 or more sputum samples (mean 15; range,10–24 samples) with corresponding clinical state and lung function data, collected over at least five years (mean 7.9; range, 5.3–10.2 years) were available per patient (S2 Table). Patient aggressiveness phenotypes were determined based on all available FEV_1_ values. Among the 24 patients, 14 had a mild disease phenotype, six were moderate, and four were severe (S1 Fig). For this analysis, moderate and severe categories were combined.

Samples from patients with a mild aggressiveness phenotype were predominantly collected during early or intermediate disease stage, while samples from patients with a moderate or severe phenotype were predominantly collected during intermediate or advanced disease stage (S2 Table). To control for bacterial community differences associated with disease stage, only samples collected during intermediate disease stage were compared. At intermediate disease stage, samples from patients with mild disease aggressiveness had a higher proportion of anaerobes compared to samples from patients with moderate or severe disease aggressiveness (0.360 and 0.321, respectively). Similar trends were observed for Shannon diversity (1.641 for mild, 1.381 for moderate/severe).

Generalized estimating equations were used to determine the impact of patient disease aggressiveness phenotype, as well as clinical state, lung function and age, on the proportion of communities consisting of anaerobic genera and Shannon diversity across all long-term samples. Overall, samples collected during exacerbation stage had significantly higher proportions of anaerobes compared to baseline (S3A Table, *P* < 0.001). The proportion of anaerobes was significantly positively associated with lung function (S3A Table, *P* < 0.001). There was a significant interaction between disease aggressiveness and age (S3A Table, *P* < 0.001). To explore this result, separate generalized estimating equation models were run for patients with mild and moderate or severe disease aggressiveness, which included clinical state, lung function and age as independent parameters. The relationship between age and the proportion of anaerobes was different for patients with mild disease aggressiveness compared with patients with moderate or severe disease aggressiveness; age was significantly positively associated with anaerobes for the former, and significantly negative associated with anaerobes for the latter (data not shown). Similar trends were observed for the relationships between clinical state, age, and disease aggressiveness, and Shannon diversity (S3B Table).

## Discussion

With this set of 631 CF sputum samples, collected from 111 patients within a single CF care center, we describe patterns of bacterial taxa prevalence and elements of bacterial community structure (dominant taxon, relative abundance of anaerobic species, and Shannon diversity) associated with patient clinical state and lung disease stage. Our findings regarding prevalence of typical CF pathogens are consistent with previous reports that describe an increase in the proportion of samples in which *Pseudomonas*, *Burkholderia*, or *Achromobacter* become the dominant taxon in airways communities with advancing disease stage [8,13]. The increased prevalence of some genera (*Haemophilus*, *Veillonella*, *Fusobacterium*, and *Porphyromonas*) in the 45+ age group may represent a survivor effect, reflecting patients with less severe disease phenotypes.

Our analysis revealed an increase in the relative abundance of anaerobes during the onset of respiratory symptoms that characterize pulmonary exacerbation, at least during the early and intermediate stages of lung disease. These observations are consistent with recent CF airway community network analyses and support the climax-attack model of CF lung infection, which describes functionally distinct airway bacterial communities during periods of clinical stability and exacerbation [6]. The attack community consists of transient bacterial populations that induce strong host immune responses, while the climax community is more stable and relatively resistant to chronic host defenses and perturbation, such as that produced by antimicrobial therapy. This model predicts that with advancing disease, climax communities, composed primarily of typical CF pathogens, become increasingly prevalent. Their resistance to antibiotic perturbation is reflected in the less pronounced shifts in community structure we observed during advanced disease stage. Our data further support a model in which advanced stage climax communities are less susceptible to influence by an attack community at exacerbation, as evidenced by the relatively minor changes in anaerobe relative abundance we observed between baseline and exacerbation states during the advanced disease stage.

We recently reported that the relative abundance of non-typical CF pathogens, including anaerobic species, was lower in samples with overall lower community diversity [5]. During the course of CF lung disease, diversity decreases both in the short term with episodic antibiotic treatment, as well as in the longer-term with progressively declining lung function. This latter decline in diversity likely results from the accumulation of intensive antibiotic exposure associated with advancing lung disease [8,14]. Examining the relative abundance of anaerobes in the context of patient clinical state and disease stage in the current study, we similarly found that both antibiotic treatment of exacerbation (in the short term) and advancing lung disease (in the long term) drive the relationship between diversity and the proportion of the community comprised of anaerobes. That is, across the large sample set included in this study, anaerobic bacteria accounted for a smaller fraction of the community during antibiotic treatment and during the advanced stage of lung disease, contributing to the decrease in community diversity observed in both conditions.

Our analysis of long-term sample sets allowed us to account for the influence of disease aggressiveness on community structure in a subset of 24 patients. Based on a comparison of only intermediate stage samples, lung communities in patients with moderate or severe disease aggressiveness appear to lose the relative proportion of anaerobes earlier compared to communities of patients with a mild disease phenotype. Generalized estimating equation analysis highlighted the importance of the interaction between disease aggressiveness and age, wherein the decline in the proportion of anaerobes with advancing age was more pronounced for patients with a moderate or severe disease aggressiveness phenotype than for patients with a mild phenotype. In other words, while the FEV_1_ term included in the generalized estimating equation model represents lung function decline independent of age, the disease aggressiveness term adds another dimension by taking into account the age during which this decline occurred. These data expand observations made in our previous work [8], where patients with a progressive disease phenotype showed decreased diversity over time and eventual dominance by a typical CF pathogen, compared to patients with a mild disease phenotype, who maintained relatively diverse airway communities. That result was found to be driven primarily by antibiotic use, rather than age or lung function. We hypothesize a similar relationship exists for the data presented here, although we did not quantify antibiotic use in this study.

The sample set employed in this study presented limitations, particularly with respect to assessing the impact of disease aggressiveness. We were unable to control for the distribution of clinical states among the available long-term samples, and as a result some patients’ samples were strongly biased with regard to clinical state (*B*,*E*,*T*,*R*) categories. For example, all samples from two patients with mild disease aggressiveness were collected at baseline, while one patient with moderate disease aggressiveness had no samples collected at baseline. The makeup of samples also changed over time for some patients. As patients with more aggressive disease phenotypes are likely receiving more frequent antibiotic therapy (i.e., more treatment courses for pulmonary exacerbations), this suggests a greater impact of antibiotic treatment on bacterial communities in airways of these patients, although the influence of antibiotic type, route, and duration was not specifically assessed. Prospectively collected, high-resolution longitudinal sample sets will be needed to examine these factors in greater detail. and to determine if and how these features of lung communities can translate to patient care.

## Conclusion

Despite the well described observation of a high degree of interpatient variability in airway bacterial community composition in CF, longitudinal analyses have identified consistent trends in the temporal dynamics of these communities. These changes include an increase in the relative abundance of anaerobic bacteria at the time of exacerbation. It is important to note, however, that fluctuations in community structure and function occur both in the short-term, coincident with changes in patients’ clinical state, as well as in the long-term, relative to advancing lung disease stage. Additionally, the nature of the short-term fluctuations in community structure occurring with changes in patients’ clinical state differ by lung disease stage. That is, the fluctuation in community structure that occurs between baseline and exacerbation at early lung disease stage differs from that occurring between baseline and exacerbation at intermediate or advanced lung disease stage. Consequently, patient clinical state and disease stage must be taken into account in interpreting results of studies assessing CF airway communities. Our results also highlight the importance of disease aggressiveness and the utility of a long-term perspective. A better appreciation of community dynamics in the context of patients’ health status is an important step in advancing our understanding of the role airway microbiota play in pulmonary exacerbations and disease progression in CF.

## Supporting information

S1 Methods Additional details describing DNA extraction, sequencing and data analyses. (DOCX)

S1 Table Generalized estimating equations. Influence of patient-specific factors on bacterial community structure.
(DOCX)

S2 Table Long-term samples. Demographics for 350 samples from 24 subjects with long-term sample sets.
(DOCX)

S3 Table Generalized estimating equations. Influence of patient disease aggressiveness phenotype on bacterial community structure.
(DOCX)

S1 Fig Aggressiveness plots. Plots depicting lung function measurements for 24 subjects with long-term sample sets. The relationship between FEV1% predicted and age separates the subjects into three categories: (A) mild (right of green line) and (B) moderate (between green and red lines) or severe (below red line).
(PDF)

S2 Fig Top genera. Prevalence of typical CF pathogens and anaerobig genera (with >1% mean relative abundance when present) by age group (A, B), clinical state (C, D), and disease stage (E, F).
(TIF)

S3 Fig Dominant genera. The proportion of samples in which the indicated genus or group of genera is dominant, stratified by lung disease stage.
(TIF)
