# Supplementary material for: Fluctuations in airway bacterial communities associated with clinical states and disease stages in cystic fibrosis
Source: PLoS One. 2018 Mar 9;13(3):e0194060. doi: 10.1371/journal.pone.0194060 (PMC5844593; doi:10.1371/journal.pone.0194060)
Supplement: S1 Methods — (DOCX) [file pone.0194060.s001.docx]

**Supporting Information**

**S1 Methods**

**DNA extraction, sequencing and data analyses**

Briefly, samples were treated with Sputolysin (EMD Chemicals, Gibbstown, NJ) and subjected to bead beating before DNA extraction using a MagNA Pure nucleic acid purification platform (Roche Diagnostics Corp., Indianapolis, IN). Pyrosequencing of the bacterial 16S ribosomal subunit (16S rRNA) gene was performed by the Human Genome Sequencing Center at Baylor College of Medicine using Roche 454-based sequencing protocols developed for the Human Microbiome Project (<http://www.hmpdacc.org/resources/tools_protocols.php>). Primer 357F (5′-CCTACGGGAGGCAGCAG-3′) modified with the addition of the 454 FLX-titanium adaptor “B” sequence (5′-CCTATCCCCTGTGTGCCTTGGCAGTCTCAG-3′) and primer 926R (5′-CCGTCAATTCMTTTRAGT-3′) modified with the addition of unique six- to eight-nucleotide barcode sequences and the 454 FLXtitanium adaptor “A” sequence (5′-CCATCTCATCCCTGCGTGTCTCCGACTCAG-3′) were used to amplify the V3, V4, and V5 hyperveriable regions. Barcode and adaptor sequences can be found at https://www.hmpdacc.org/hmp/doc/HMP_MDG_454_16S_Protocol.pdf.

A subset of sequencing data from a previous dataset [1] was analyzed independently from the Operational Taxonomic Units (OTU) clustering steps forward. OTUs were assigned at a 3% dissimilarity cutoff, presumptively to species level. OTUs were binned into genera by aligning the DNA sequences to the Ribosomal Database Project [2] training set containing 9,665 bacterial and 384 archaeal 16S rRNA gene sequences. To identify OTUs that were unclassified at the genus level, a representative sequence was generated that contained the highest homology to all other sequences in the OTU. These representative sequences were identified by using a BLAST search against the NCBI nucleotide collection (nr/nt) database and reporting the best match.

**References**

1. Mahboubi MA, Carmody LA, Foster BK, Kalikin LM, VanDevanter DR, LiPuma JJ. Culture-based and culture-independent bacteriologic analysis of cystic fibrosis respiratory specimens. J Clin Microbiol. 2016;54: 613-619.
2. Cole JR, Wang Q, Cardenas E, Fish J, Chai B, Farris RJ, et al. The Ribosomal Database Project: improved alignments and new tools for rRNA analysis. Nucleic Acids Res. 2009;37: D141–D145.
