# Supplementary material for: Fluctuations in airway bacterial communities associated with clinical states and disease stages in cystic fibrosis
Source: PLoS One. 2018 Mar 9;13(3):e0194060. doi: 10.1371/journal.pone.0194060 (PMC5844593; doi:10.1371/journal.pone.0194060)
Supplement: S1 Table — Influence of patient-specific factors on bacterial community structure. (DOCX) [file pone.0194060.s002.docx]

**S1 Table.** **Generalized estimating equations:** influence of patient-specific factors on bacterial community structure**.**

**A**

| Parameter | B | Std. Error | 95% Wald Confidence Interval | | Hypothesis Test | |
| --- | --- | --- | --- | --- | --- | --- |
|  |  |  | Lower | Upper | Wald Chi-Square | Sig. |
| Clinical State  **B**aseline | reference |  |  |  |  |  |
| **E**xacerbation | .170 | .0324 | .106 | .233 | 27.450 | <.001 |
| **T**reatment | -.040 | .0532 | -.144 | .064 | .564 | .453 |
| **R**ecovery | .034 | .0474 | -.058 | .127 | .527 | .468 |
| FEV_1_ | .008 | .0012 | .005 | .010 | 43.374 | <.001 |
| Age | -.003 | .0027 | -.008 | .002 | 1.443 | .230 |

**B**

| Parameter | B | Std. Error | 95% Wald Confidence Interval | | Hypothesis Test | |
| --- | --- | --- | --- | --- | --- | --- |
|  |  |  | Lower | Upper | Wald Chi-Square | Sig. |
| Clinical State  **B**aseline | reference |  |  |  |  |  |
| **E**xacerbation | .154 | .0343 | .087 | .221 | 20.115 | <.001 |
| **T**reatment | -.334 | .0572 | -.446 | -.221 | 33.990 | <.001 |
| **R**ecovery | -.050 | .0425 | -.133 | .033 | 1.383 | .240 |
| FEV_1_ | .006 | .0009 | .004 | .008 | 40.808 | <.001 |
| Age | -.008 | .0028 | -.013 | -.002 | 7.695 | .006 |

Generalized estimating equations estimating the effects of clinical state (*B,E,T,R*), lung function (FEV_1_), and age on (A) cumulative relative abundance of anaerobic genera (*Actinomyces, Fusobacterium, Gemella, Granulicatella, Porphyromonas*, *Prevotella,* *Rothia*, *Streptococcus* and *Veillonella* spp), and (B) Shannon diversity.
