# Supplementary material for: Fluctuations in airway bacterial communities associated with clinical states and disease stages in cystic fibrosis
Source: PLoS One. 2018 Mar 9;13(3):e0194060. doi: 10.1371/journal.pone.0194060 (PMC5844593; doi:10.1371/journal.pone.0194060)
Supplement: S2 Table — Demographics for 350 samples from 24 subjects with long-term sample sets. (DOCX) [file pone.0194060.s003.docx]

**S2 Table. Long-term samples.**

|  | | Disease Aggressiveness Phenotype | |
| --- | --- | --- | --- |
|  |  | Mild  n=178 samples,  14 subjects | Moderate/Severe  n=172 samples,  10 subjects |
| Mean age, years (range) |  | 33 (20-54) | 22 (11-31) |
| Clinical State (n)  Disease Stage (n) | **B**aseline  **E**xacerbation  **T**reatment  **R**ecovery  Early (FEV_1_ > 70) | 101  30  20  27  88 | 54  33  50  35  7 |
|  | Intermediate (40 ≤ FEV_1_ ≤ 70) | 79 | 90 |
|  | Advanced (FEV_1_ < 40) | 11 | 75 |

Demographics for 350 samples from 24 subjects with long-term sample sets.
