# Supplementary material for: Fluctuations in airway bacterial communities associated with clinical states and disease stages in cystic fibrosis
Source: PLoS One. 2018 Mar 9;13(3):e0194060. doi: 10.1371/journal.pone.0194060 (PMC5844593; doi:10.1371/journal.pone.0194060)
Supplement: S3 Table — Influence of patient disease aggressiveness phenotype on bacterial community structure. (DOCX) [file pone.0194060.s004.docx]

**S3 Table. Generalized estimating equations:** influence of patient disease aggressiveness phenotype on bacterial community structure**.**

**A**

| Parameter | B | Std. Error | 95% Wald Confidence Interval | | Hypothesis Test | | |
| --- | --- | --- | --- | --- | --- | --- | --- |
|  |  |  | Lower | Upper | Wald Chi-Square | df | Sig. |
| (Intercept) | -2.525 | .2430 | -3.001 | -2.048 | 107.964 | 1 | <.001 |
| Clinical State  **B**aseline | reference |  |  |  |  |  |  |
| **E**xacerbation | .317 | .0445 | .230 | .404 | 50.859 | 1 | <.001 |
| **T**reatment | -.102 | .0571 | -.214 | .010 | 3.206 | 1 | .073 |
| **R**ecovery | .030 | .0499 | -.068 | .128 | .360 | 1 | .548 |
| FEV_1_ | .011 | .0019 | .007 | .014 | 31.431 | 1 | <.001 |
| Age | .023 | .0037 | .016 | .030 | 39.605 | 1 | <.001 |
| Aggressiveness  Mild | reference |  |  |  |  |  |  |
| Moderate/Severe | 2.010 | .2548 | 1.511 | 2.510 | 62.250 | 1 | <.001 |
| Mild*Age | reference |  |  |  |  |  |  |
| Moderate/Severe*Age | -.070 | .0084 | -.087 | -.054 | 69.159 | 1 | <.001 |
| (Scale) | .055 |  |  |  |  |  |  |

**B**

| Parameter | B | Std. Error | 95% Wald Confidence Interval | | Hypothesis Test | | |
| --- | --- | --- | --- | --- | --- | --- | --- |
|  |  |  | Lower | Upper | Wald Chi-Square | df | Sig. |
| (Intercept) | -.108 | .1595 | -.420 | .205 | .456 | 1 | .500 |
| Clinical State  **B**aseline | reference |  |  |  |  |  |  |
| **E**xacerbation | -.218 | .0944 | -.403 | -.033 | 5.350 | 1 | .021 |
| **T**reatment | .087 | .0633 | -.038 | .211 | 1.866 | 1 | .172 |
| **R**ecovery | -.029 | .0580 | -.143 | .085 | .252 | 1 | .616 |
| FEV_1_ | .003 | .0014 | .000 | .006 | 4.934 | 1 | .026 |
| Age | .009 | .0023 | .005 | .014 | 16.167 | 1 | <.001 |
| Aggressiveness  Mild | reference |  |  |  |  |  |  |
| Moderate/Severe | .846 | .2077 | .439 | 1.253 | 16.595 | 1 | <.001 |
| Mild*Age | reference |  |  |  |  |  |  |
| Moderate/Severe*Age | -.036 | .0088 | -.053 | -.018 | 16.572 | 1 | <.001 |
| (Scale) | .055 |  |  |  |  |  |  |

Generalized estimating equations estimating the effects of clinical state (*B,E,T,R*), lung function (FEV_1_), age, and disease aggressiveness phenotype on (A) cumulative relative abundance of anaerobic genera (*Actinomyces, Fusobacterium, Gemella, Granulicatella, Porphyromonas*, *Prevotella,* *Rothia*, *Streptococcus* and *Veillonella* spp), and (B) Shannon diversity.
