# Supplementary material for: Fluctuations in airway bacterial communities associated with clinical states and disease stages in cystic fibrosis
Source: PLoS One. 2018 Mar 9;13(3):e0194060. doi: 10.1371/journal.pone.0194060 (PMC5844593; doi:10.1371/journal.pone.0194060)

S1 Fig. Aggressiveness plots. Plots depicting lung function measurements for 24 subjects with long-term sample sets. The relationship between FEV1 % predicted and age separates the subjects into three categories: (A) mild (right of green line) and (B) moderate (between green and red lines) or severe (below red line).

A

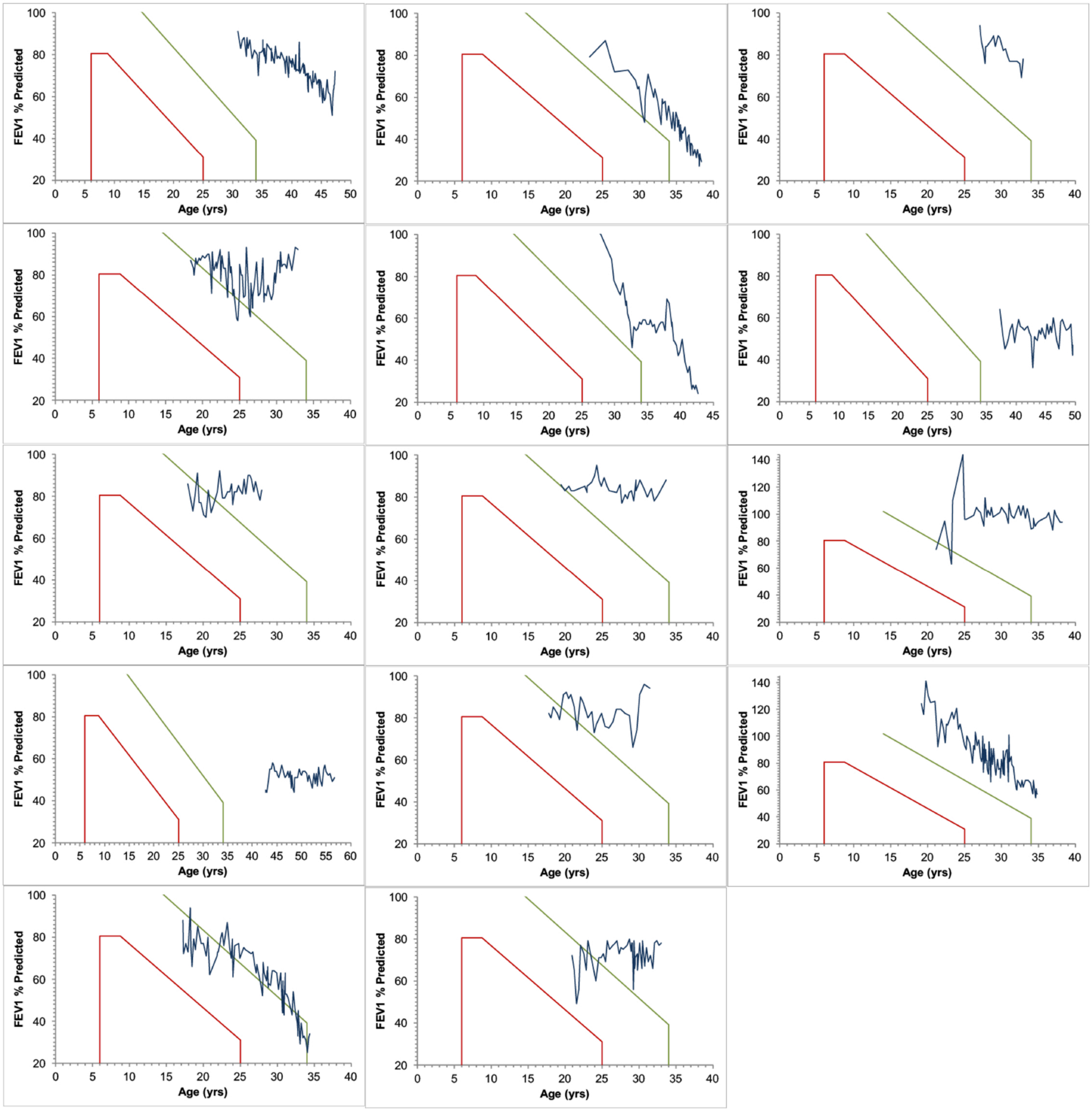

B

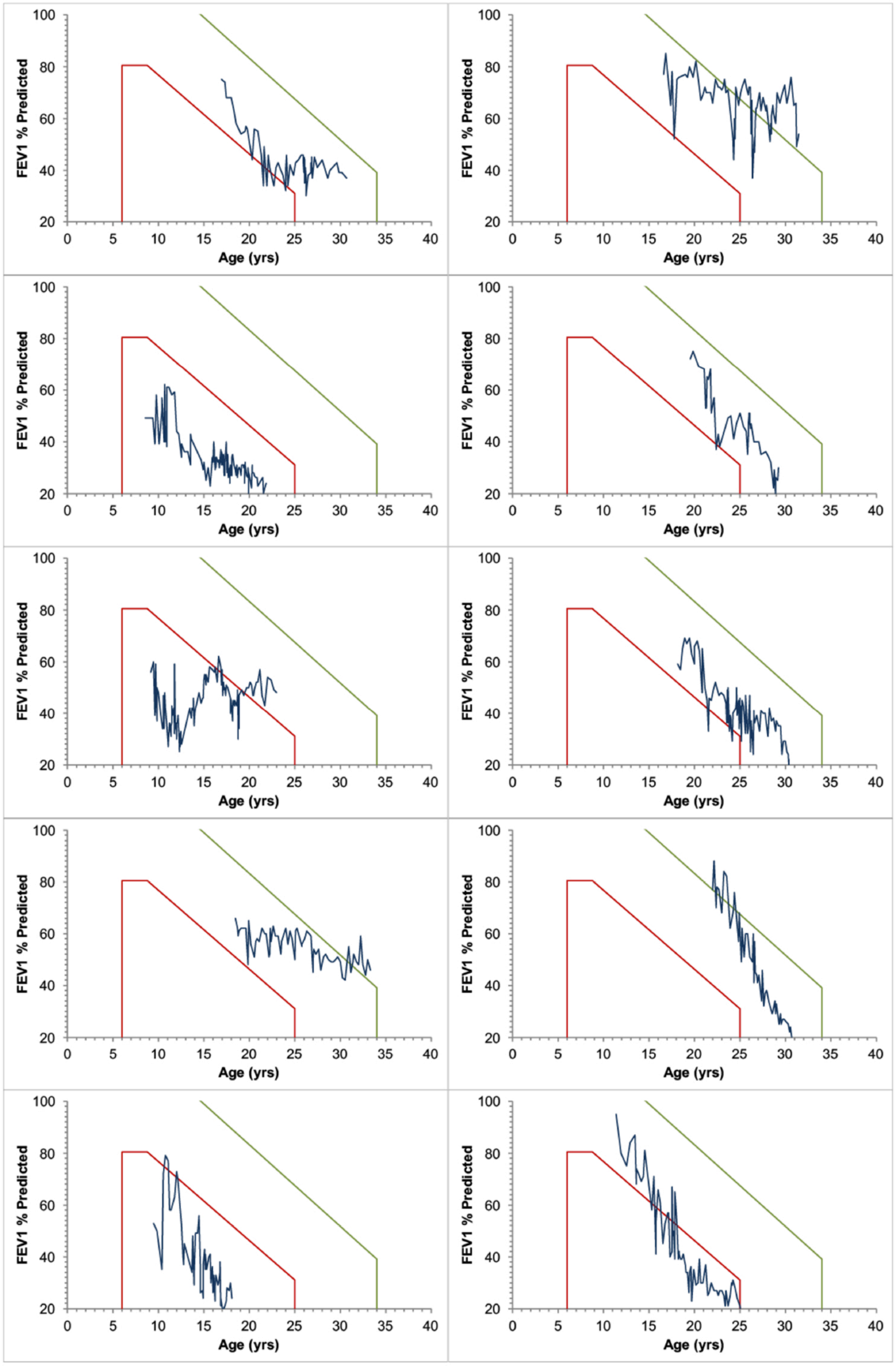

Supplement: S1 Fig — Plots depicting lung function measurements for 24 subjects with long-term sample sets. The relationship between FEV1% predicted and age separates the subjects into three categories: (A) mild (right of green line) and (B) moderate (between green and red lines) or severe (below red line). (PDF) [file pone.0194060.s005.pdf]
