# Supplementary figures and images for: Fluctuations in airway bacterial communities associated with clinical states and disease stages in cystic fibrosis
Source: PLoS One. 2018 Mar 9;13(3):e0194060. doi: 10.1371/journal.pone.0194060 (PMC5844593; doi:10.1371/journal.pone.0194060)

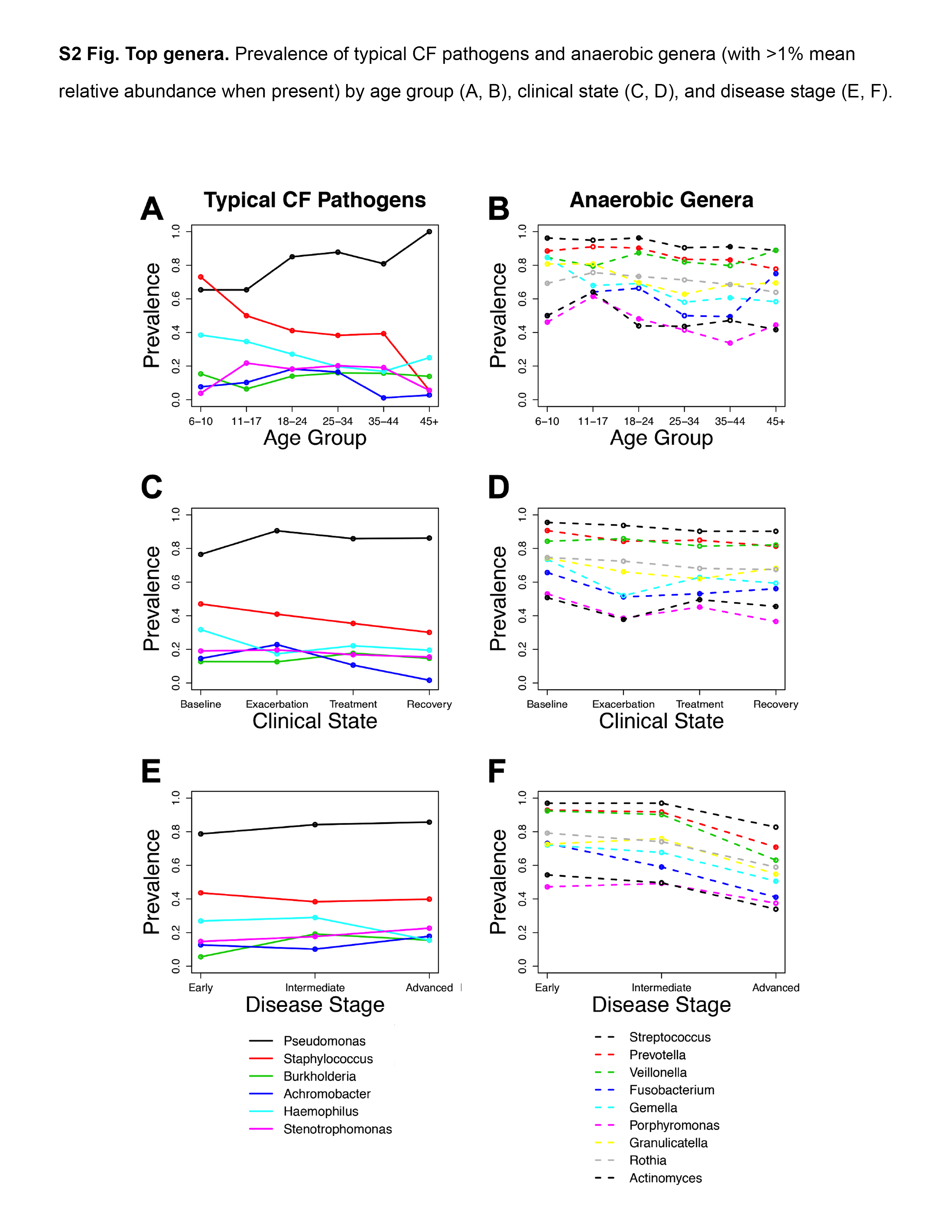

Supplement: S2 Fig — Prevalence of typical CF pathogens and anaerobig genera (with >1% mean relative abundance when present) by age group (A, B), clinical state (C, D), and disease stage (E, F). (TIF) [file pone.0194060.s006.tif]

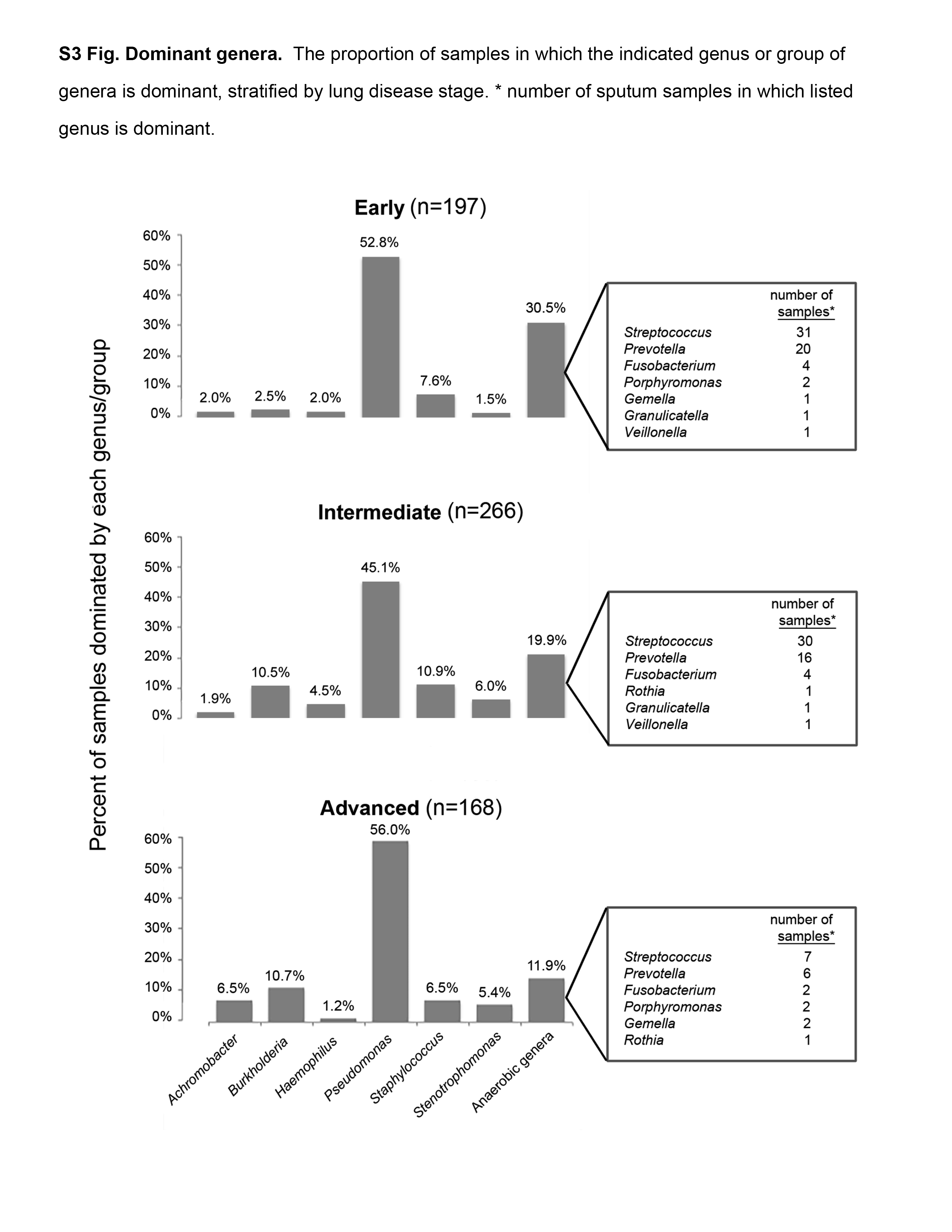

Supplement: S3 Fig — The proportion of samples in which the indicated genus or group of genera is dominant, stratified by lung disease stage. (TIF) [file pone.0194060.s007.tif]
